# Bisphenol A affects early bovine embryo development and metabolism that is negated by an oestrogen receptor inhibitor

**DOI:** 10.1038/srep29318

**Published:** 2016-07-07

**Authors:** Bom-Ie Choi, Alexandra J. Harvey, Mark P. Green

**Affiliations:** 1 School of BioSciences, University of Melbourne, Parkville, Melbourne, VIC 3010, Australia

## Abstract

Increasing evidence supports an association between exposure to endocrine disruptors, such as the xenoestrogen bisphenol A (BPA), a commonly used plasticiser, and the developmental programming of offspring health. To date however animal studies to investigate a direct causal have mainly focussed on supra-environmental BPA concentrations, without investigating the effect on the early embryo. In this study we investigated the effect of acute BPA exposure (days 3.5 to 7.5 post-fertilisation) at environmentally relevant concentrations (1 and 10 ng/mL) on *in vitro* bovine embryo development, quality and metabolism. We then examined whether culturing embryos in the presence of the oestrogen receptor inhibitor fulvestrant could negate effects of BPA and 17β-oestradiol (E2). Exposure to BPA or E2 (10 ng/mL) decreased blastocyst rate and the percentage of transferrable quality embryos, without affecting cell number, lineage allocation or metabolic gene expression compared to untreated embryos. Notably, blastocysts exposed to BPA and E2 (10 ng/mL) displayed an increase in glucose consumption. The presence of fulvestrant however negated the adverse developmental and metabolic effects, suggesting BPA elicits its effects via oestrogen-mediated pathways. This study demonstrates that even acute exposure to an environmentally relevant BPA concentration can affect early embryo development and metabolism. These may have long-term health consequences on an individual.

Endocrine disruptors have begun to receive greater attention in the field of reproductive biology and developmental programming^1^,^2^,^3^,^4^. Bisphenol A (BPA) is one of the most studied endocrine disruptors and also one of the highest volume chemicals produced worldwide^5^,^6^,^7^. This synthetic oestrogen (xenoestrogen) is found in a wide range of everyday products, such as soft plastic bottles, the lining of aluminum food cans and the coating of receipts^5^. Hence, it has now become virtually impossible for humans, and indeed many other species, to avoid daily exposure to BPA. Despite this, very little is known of the exact mechanisms of action and the concentration, as well as timing and length of exposure, that can negatively affect the metabolism and reproductive function of an individual.

BPA is known to bind competitively to different types of oestrogen receptors (ERs), including ERα and ERβ, with a higher affinity for ERβ^8^. BPA can also act via oestrogen-independent pathways, for example BPA exposure is positively correlated with androgen levels^9^ and also inhibits thyroid hormone action by acting as an antagonist^10^. However, the mechanism of action by which BPA exerts its effects, specifically via secondary messenger pathways to cause alterations in cellular physiology, or in terms of early developmental exposure, is not yet fully understood.

In the human population, reports confirm the presence of BPA in over 95% of urine samples^11^,^12^. Recent epidemiological studies have now identified a strong correlation between high urinary BPA concentrations and a higher incidence of serious health complications, such as cardiovascular disease^13^,^14^, obesity^15^,^16^ and type II diabetes^17^. These studies suggest that BPA exposure may be causal or contribute to the incidence and severity of diseases with serious long-term health implications. Evidence from rodent studies supports human epidemiological data, with a negative correlation between BPA and adult metabolism, specifically glucose homeostasis, insulin resistance, as well as metabolic perturbations evident in offspring exposed during gestation^18^,^19^,^20^,^21^.

BPA is present and has been measured in many human fluids and tissues associated with reproduction; follicular fluid (1.5 to 2.4 ng/mL), amniotic fluid (1 to 17 ng/mL), placental tissue (11.2 ng/mL) and breast milk (0.28 to 1.9 ng/mL)^22^,^23^, as well as similar concentrations being determined in the reproductive fluids and tissues of domestic species^24^,^25^. Together these data establish environmentally relevant *in vivo* BPA concentrations to be in the range of 0.5 to 15 ng/mL. The presence of BPA in reproductive tissues has negative effects, such as decreased spermatogenesis and increased aneuploidy in mice^26^,^27^, as well as poor reproductive outcomes. Notably, higher urinary BPA levels in human IVF patients are associated with lower numbers of oocytes, as well as a reduction in the percentage of normally fertilised oocytes^28^. In addition, experimental animal studies have identified that BPA administrated orally or via injection, generally at supra-environmentally relevant concentrations, can affect numerous aspects of normal reproductive function, including gametogenesis^26^,^29^, timing of puberty^30^ and development of both female and male reproductive tracts^6^.

Variation in the timing, length and dose of BPA exposure during pregnancy in animals has begun to be studied. These studies have identified that gestational exposure to a very high BPA concentration (100 mg/kg body weight) severely delays development to the blastocyst stage, completely inhibits implantation^31^ and decreases the number of live offspring born in mice^32^, although studies often fail to measure the exact *in vivo* BPA concentration to which embryos are exposed *in utero*. While chronic exposure to supra-environmentally relevant concentrations demonstrate BPA can have dramatic effects on reproductive function, only three studies have examined the effects of more environmentally relevant concentrations at specific stages of embryo development using an *in vitro* production system^33^,^34^,^35^. BPA exposure (30 ng/mL) during bovine oocyte maturation resulted in increased abnormalities in MII oocytes and a subsequent decrease in embryo development rates, as well as increased DNA fragmentation^33^,^34^. Similarly, Takai and colleagues^35^ demonstrated decreased mouse embryo development to the blastocyst stage following a two-day exposure to BPA (equivalent to 22.8 μg/mL). Surprisingly, to date, metabolic studies of BPA exposed embryos have not been performed. This is despite the growing body of evidence that clearly show modulation of the early embryonic environment affects the long-term health of offspring^36^,^37^,^38^. We postulate that one critical developmental window for BPA exposure would be during early embryo development, particularly during the developmentally sensitive window from embryonic genome activation (EGA) through to the blastocyst stage, as has been established for a range of environmental factors responsible for affecting the phenotype of offspring^36^,^39^. In the current study we utilise the well-defined bovine *in vitro* embryo production system to investigate this theory. We selected the bovine embryo as it is physiologically similar to the human embryo and has many advantages over the use of rodents for studying the impacts of endocrine disruptors^40^,^41^,^42^.

To date no study has investigated the potentially subtle effects of environmentally-relevant BPA concentrations on early embryonic development and metabolism, which may have considerable implications for the long-term health of offspring. The aim of this study was to investigate the effect of acute exposure (post-EGA, days 3.5 to 7.5 post-fertilisation) of environmentally relevant BPA concentrations (1 and 10 ng/mL) on *in vitro* bovine embryo development and metabolism. To explore this question, we assessed development rates, embryo quality, cell lineage allocation, and carbohydrate metabolism of individual embryos, as well as changes in the expression of metabolic and oestrogen-signaling pathway genes. We also examined the mechanism of BPA action, via oestrogen-mediated pathways, with the addition of the oestrogen-receptor (ER) inhibitor fulvestrant to culture media, in order to mitigate the potential detrimental effects of BPA on the early embryo.

## Materials and Methods

### Oocyte collection and embryo culture

All reagents were sourced from Sigma-Aldrich unless otherwise stated. The methods for oocyte collection, maturation, fertilisation and *in vitro* culture to the 8-cell stage have been previously described^43^,^44^. Briefly, bovine cumulus-oocyte-complexes (COC) were collected from antral follicles from abattoir-derived ovaries in HEPES-buffered tissue culture medium-199 (TCM 199) supplemented with 10% fetal calf serum (FCS; Life Technologies) and 4 mg/mL fatty acid-free bovine serum albumin (FAF-BSA; MP BioMedical). COCs with an intact cumulus vestment and homogenous cytoplasm were selected and washed in HEPES-TCM-199 supplemented with 50 mg/mL kanamycin, 50 mg/mL heparin and 4 mg/mL FAF-BSA and subsequently washed twice in HEPES-TCM199. COCs were matured in groups of 50 for 24 h in bicarbonate-buffered TCM199 supplemented with 0.1 IU/mL hCG (Merck Serono), 1 IU/mL recombinant human FSH (Organon), 1 μM cysteamine, 10% (w/v) FCS, and 4 mg/mL FAF-BSA under embryo-grade mineral oil in Nunc 4-well dishes (Thermo Scientific) at 38.5 °C under 5% CO_2_ and 20% O_2_. Following *in vitro* maturation, oocytes (n = 20 per well) were fertilised with sperm from a single sire (Semex Pty Ltd) at a final concentration of 1 × 10^6^/mL in *in vitro* fertilisation medium supplemented with amino acids (50 μL) under mineral oil in a 96-well plate (Thermo Scientific) at 38.5 °C under 5% CO_2_ in air. Presumptive zygotes were recovered after 23 h, cumulus cells manually stripped and resultant embryos transferred into Nunc 4-well dishes (n = 20 per well) containing synthetic oviductal fluid (SOF) medium supplemented with amino acids and 4 mg/mL BSA (500 μL per well). Embryos were then cultured in a modular incubator at 38.5 °C under 5% O_2_ and 6% CO_2_ for 60 h.

### Experiment 1: Effect of BPA exposure on embryo development and quality

The aim of this experiment was to investigate the effects of environmentally relevant BPA concentrations, as determined from concentrations measured in reproductive fluids and tissues^22^,^23^, on pre-implantation embryo development and quality. Equivalent 17β-oestradiol (E2) treatment groups were also included at the same concentrations as BPA in each culture, as a reference, since BPA can act via oestrogen-mediate pathways. Only embryos consisting of eight or more cells were selected to proceed with the experiment. Embryos were randomly allocated to one of five treatments; 0 ng/mL (control), 1 ng/mL BPA (low BPA), 10 ng/mL BPA (high BPA), 1 ng/mL E2 (low E2) or 10 ng/mL E2 (high E2) in L-SOF medium supplemented with amino acids and 8 mg/mL BSA (500 μL). Embryos (n = 15 per well) were cultured in a Nunc 4-well dish, without an oil overlay, to which Milli-Q water (purified at 18.2 MΩ/cm at 25 °C by a Milli-Q-Elix® purification unit (Merck Millipore)) was placed between the 4-wells to prevent medium evaporation in a modular incubator at 38.5 °C under 5% O_2_ and 6% CO_2_ for 96 h. On day 7.5 post-IVF, embryo developmental stage and quality grade were assessed (grades 1 (good) to 3 (poor) as previously defined^45^. Blastocyst rates were calculated (per culture) as the percentage of embryos that reached the blastocyst stage from the number of 8^+^ cell-stage embryos assigned to treatments. Following developmental assessment, blastocysts were either stained (differential cell stain, see below) or snap-frozen and stored at −80 °C for qRT-PCR analysis.

### Differential cell staining

The number of cells in the inner cell mass (ICM) and the trophectoderm (TE) of expanded and hatched blastocysts was determined using differential staining on day 7.5 post-IVF, based on previously published methods^46^. Briefly, embryos were placed in 0.5% (w/v; 10 μL) pronase in GMOPS (Vitrolife) to remove the zona pellucida, and then washed in GMOPS-PLUS (Vitrolife) for 5 min. Embryos were subsequently incubated in 10 μL of 10 mM trinitrobenzenesulfonic acid (TNBS) in 4 mg/mL polyvinyl pyrollidone (PVP) in simple-G1 medium (Vitrolife; G1-PVP) for 20 min, washed twice in GMOPS-PLUS then transferred to 0.1 mg/mL anti-2,4 dinitrophenol-G1-PVP (10 μL) for 10 min. Following a further wash in GMOPS-PLUS, embryos were transferred to 10 μL drops of 10% v/v guinea pig serum in 25 mg/mL propidium iodide in GMOPS for 1 min, and then placed into 10 μL drops of 0.1 mg/mL bisbenzimide for 20 min. Embryos were individually mounted in 100% glycerol on a glass slide under a coverslip. Embryos were viewed under fluorescent light using a Nikon Eclipse TS100 microscope equipped with a mercury lamp (Olympus) and images of embryos were taken using the Nikon Digital Sight DS-L2 (Nikon). The number of cells in the inner cell mass (ICM) and the trophectoderm (TE) were counted using the cell-counter tool in ImageJ software (National Institute of Health).

### Metabolic microfluorescence assays and total cell stain

In each culture a cohort of five to ten morula-stage embryos from each treatment were randomly selected on day 6.5 and allocated for individual culture in a 4 μL drop of L-SOF medium supplemented with amino acids, 8 mg/mL BSA and the corresponding treatment. Embryos were cultured for 24 h prior to the measurement of glucose consumption and lactate production from spent medium by microfluorescence assays adapted from previous reports^47^,^48^,^49^. For each treatment, embryos were cultured along with two empty drops of their respective medium per replicate, to be used as unspent, blank controls. On day 7.5, embryos were assessed for development and quality, before being subjected to total cell staining (0.1 mg/mL bisbenzimide in 10% v/v ethanol, 90% v/v GMOPS for 20 min at 37 °C, n > 22 in each group) and individually mounted in 100% glycerol on a glass slide under a coverslip. For each individual spent medium drop, 1 μL was added to either the glucose or lactate cocktails, as previously described^47^ for enzymatic reactions in which fluorescent molecules NADPH and NADH are produced respectively. The fluorescence of NADPH or NADH was quantified as an indirect measure of glucose and lactate respectively. To determine actual consumption of glucose, the sample measurement is subtracted from the mean blank measurement. To determine actual production of lactate, the mean blank measurement is subtracted from the sample measurement. Metabolite use was expressed per cell per hour to account for differences between individual embryos in total cell number.

### RNA isolation and cDNA synthesis

Total RNA was isolated from five independent biological pools of 20 unhatched (early blastocyst, blastocyst and expanded blastocyst stage) embryos frozen in lysis buffer using the Absolutely RNA Nanoprep Kit (Agilent Technologies) as per the manufacturer’s (Agilent Technologies) instructions. RNA was treated with DNase (Agilent Technologies) to minimize contamination. Resultant RNA was eluted with 10 μL of elution buffer and diluted to 12 μL with nuclease free water (Life Technologies). The cDNA was synthesized from 20 μL of RNA using SuperScript III (Life Technologies) and random primers (Promega) according to the manufacturer’s instructions (Life Technologies).

### Quantitative RT-PCR of oestrogen-mediated and metabolic pathway genes

Changes were investigated in the expression of oestrogen signaling pathway (*oestrogen receptor alpha and beta*, *ERα* and *ERβ*), glucose transport (*Glucose transporters*, *SLC2A1*, *SLC2A3*, *SLC2A4*; also known as *GLUT1*, *GLUT3 and GLUT4*), glycolysis (*Glucose-6-phosphate isomerase*, *GPI*), pentose phosphate pathway (*Glucose-6-phosphate dehydrogenase*, *G6PD* and *6-phosphogluconolactonase*, *PGLS*), Krebs cycle (*Aconitase 2*, *ACO2*) and mitochondrial biogenesis (*Mitochondrial transcription factor A*, *TFAM*) genes. Primers were designed using PrimerExpress3 (Life Technologies) or selected from the literature, as outlined in Table 1, and synthesized by Geneworks. All PCR reactions were performed in a ViiA7 real-time PCR machine (Life Technologies) using SYBR Green PCR master mix (Life Technologies). The following thermal profile was used: 95 °C for 10 min, 95 °C for 15 s then 60 °C for 1 min, cycled 40 times; 72 °C for 5 min. Each sample was run in triplicate with minus RT and no template (water) controls. The efficiency of three housekeeping genes (ribosomal protein L19 (*RPL19*), *β-Actin* and *18S rRNA*) were tested at different dilutions and in the presence or absence of BPA or E2 to confirm the most appropriate housekeeper for quantitation. The relative expression of each gene was calculated using the delta delta CT method and Q-Gene software^50^, normalized to 18S rRNA.

### Experiment 2: Effects of the oestrogen-receptor (ER) inhibitor fulvestrant on embryo development and metabolism

In a separate study, to determine whether BPA acts via ER mediated pathways, 8^+^ cell embryos were cultured in the presence or absence of the ER inhibitor (fulvestrant; Sigma-Aldrich) for 96 h, as described above, in the presence or absence of BPA (10 ng/mL) or oestradiol 17β (E2, 10 ng/mL), with or without fulvestrant. A concentration of 10 ng/mL fulvestrant was selected based on a previous embryo study^35^. Embryos were randomly allocated to one of six treatment groups: control, BPA, E2, fulvestrant (FULV), BPA + FULV and E2 + FULV, and subsequently cultured until day 7.5 post-IVF when blastocyst development rates and quality were assessed as described above. Blastocysts were then subjected to differential cell staining. Similar to *Experiment 1*, a cohort of morula-stage embryos from each treatment group was randomly selected on day 6.5 for metabolic assessment, followed by total cell staining (n > 25 in each group), as described above.

### Statistical analysis

Day 7.5 developmental data, including blastocyst rate, pre- and post-expanded stage blastocyst percentages, and embryo quality were arc sine transformed prior to analysis to account for differences in the number of 8^+^ cells placed into each treatment each culture. All data, including gene expression data, were tested for normality prior to analysis. The effect of BPA and E2 on development, cell number (total cell, differential cell allocation and ratios), as well as metabolic and gene expression data were analysed by ANOVA using a PROC MIXED procedure employing a Tukey’s *post hoc* analysis to identify differences between all groups. Culture replicate was included in the model as a random factor where appropriate. All analyses were performed in SAS version 9.2 (SAS Institute). Data are presented as mean ± SEM unless otherwise stated. Significance was determined at the level of *P* < 0.05.

## Results

### Oocyte numbers, fertilisation and development rates to day 3.5

Approximately 9,800 bovine oocytes underwent *in vitro* maturation and fertilisation in 28 cultures with a mean fertilisation rate of 92%. On average, 56% of the matured oocytes reached the 8^+^ cell stage on day 3.5 of development and an overall total of 2,850 and 1,365 8^+^ cell stage embryos in total were allocated into treatment groups for *Experiment 1* and *2* respectively.

### Experiment 1: BPA negatively impacts embryo development and quality

**Figure 1:**
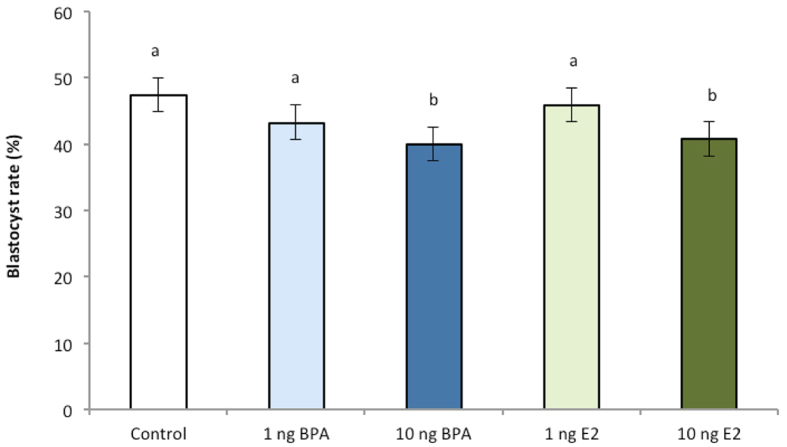
The effect of BPA and E2 supplementation (1 and 10 ng/mL) from day 3.5 to 7.5 of culture on day 7.5 blastocyst rate. n ≥ 495 8^+^ cell stage embryos per treatment across 22 biological replicates. Data are presented as mean ± s.e.m. Different superscript letters denote *P < 0.05.*

A minimum of 495 8^+^ cell stage embryos were assigned to each treatment group (control n = 495; low BPA n = 555; high BPA n = 660; low E2 n = 525; high E2 n = 615). Acute (96-hour) exposure to 10 ng/mL BPA, a high but still environmentally relevant concentration of BPA, decreased the percentage of embryos reaching the blastocyst stage compared to the control group (*P* < 0.05; Fig. 1). A significant decrease in development was also evident in the high E2 treatment group (*P* = 0.05). No differences were evident in development between all other treatment groups (*P* > 0.1). Analysis of blastocyst development stage on day 7.5 determined no differences between groups in the proportion of 8^+^ cell stage embryos that developed to either pre-expanded (early blastocysts and blastocysts) or post-expanded (≥expanded blastocysts) stages.

**Figure 2:**
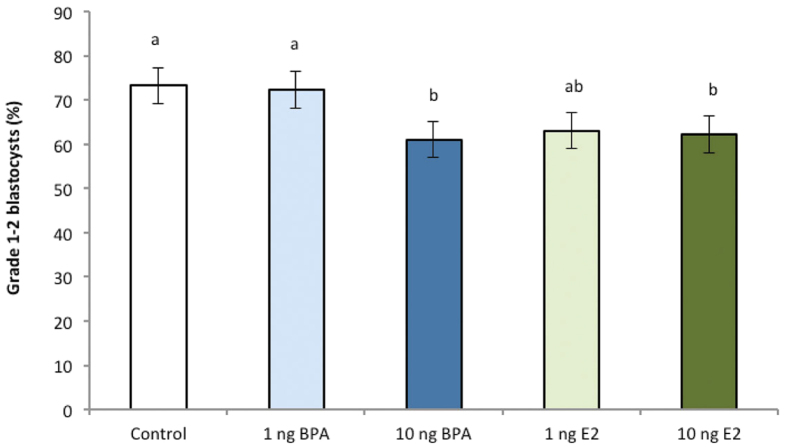
Effect of BPA or E2 supplementation (1 and 10 ng/mL) on the percentage of grade 1 and 2 (transferrable quality) day 7.5 blastocysts. n ≥ 495 8^+^ cell stage embryos per treatment across 22 biological replicates. Data are means ± s.e.m. Different superscript letters denote *P* < 0.05.

In terms of quality, an approximate 10% decrease in the percentage of transferrable quality (grades 1–2) blastocysts was detected in embryos exposed to high BPA, as well as low E2 and high E2 (*P* < 0.05) in comparison to the control and low BPA-treated embryos (Fig. 2). No differences were evident between other treatment groups (*P* > 0.1). Thus, when considered with the marked reduction in blastocyst stage embryos, an overall 20% decrease in transferrable grade embryos in the high BPA and high E2 groups was evident compared with the untreated group.

### Experiment 1: BPA effects on cell number and lineage allocation

Differential staining of day 7.5 post-expanded blastocysts demonstrated that neither BPA nor E2 supplementation altered inner cell mass (ICM), trophectoderm (TE), or total cell numbers (*P* > 0.1; Table 2). Consequently, no significant change in the ratio of ICM to TE or the percentage of ICM cells was determined (*P* > 0.1).

### Experiment 1: BPA effects on embryo metabolism

**Figure 3:**
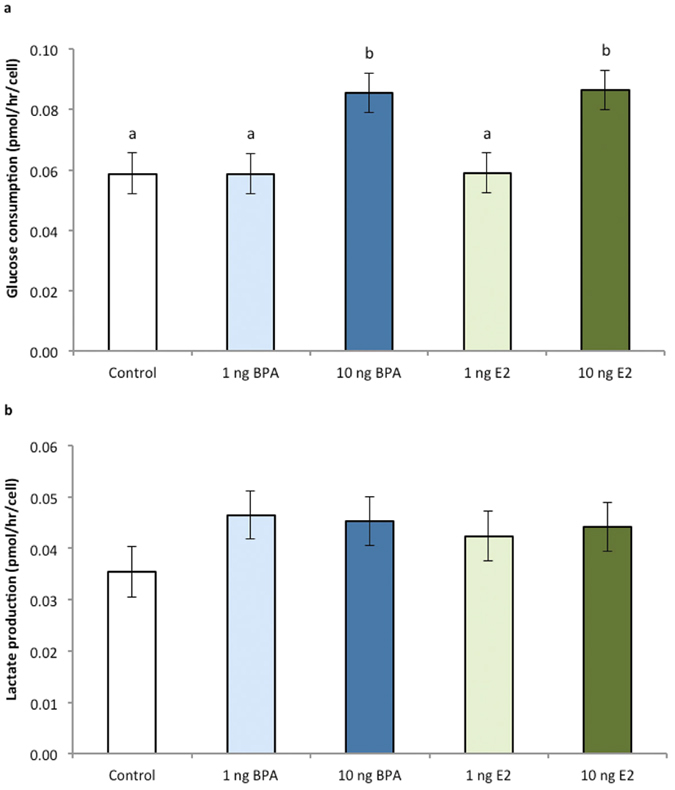
Effect of BPA or E2 supplementation (1 and 10 ng/mL) on (**a**) glucose consumption (pmol/h/cell) and (**b**) lactate production of embryos (pmol/h/cell). n ≥ 30 blastocysts per treatment across nine biological replicates. Data are mean ± s.e.m. Different superscript letters denote *P* < 0.05.

Analysis of glucose consumption and lactate production of individual blastocysts on both a per embryo, and a per cell basis, revealed a significant increase in glucose consumption in embryos exposed to high BPA and high E2 (*P* < 0.05) compared to control, low BPA and low E2-treated embryos (Fig. 3a). In contrast to glucose consumption, no change in lactate production was observed following exposure to either BPA or E2 between any groups (Fig. 3b), although the pattern of higher lactate production in the high BPA and high E2 groups followed that observed for glucose consumption.

### Experiment 1: BPA effects on expression of oestrogen-mediated and metabolic genes

**Figure 4:**
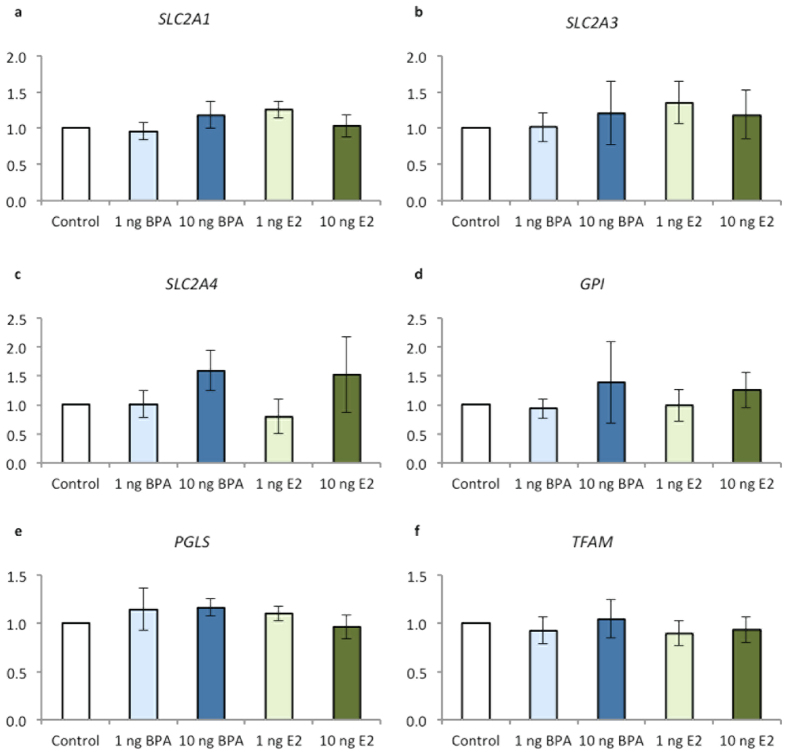
Effect of BPA or E2 supplementation (1 and 10 ng/mL) on (**a**) *Glucose transporter 1 (SLC2A1)*, (**b**) *Glucose transporter 3 (SLC2A3)*, (**c**) *Glucose transporter 4 (SLC2A4)*, (**d**) Glucose-6-phosphate isomerase (GPI), (**e**) *6-phosphogluconolactonase (PGLS)* and (**f**) *Mitochondrial transcription factor A (TFAM)* gene expression normalised to the expression of 18S and control bovine blastocysts using real-time quantitative PCR. Data are mean ± s.e.m. n = 4 replicates of 20 pooled blastocysts per treatment.

Neither BPA nor E2 supplementation significantly altered the expression of genes involved in oestrogen signaling pathways (*ERα* and *ERβ*), glucose transport (*SLC2A1*, *SLC2A3* and *SLC2A4*), glycolysis (*GPI*), the pentose phosphate pathway (*G6PD*, *PGLS*), Krebs cycle (*ACO2*) or mitochondrial biogenesis (*TFAM*) between any of the treatments groups (*P* > 0.1 for each gene; data for selected genes shown in Fig. 4). Elevated expression, although non-significant was evident in both the high BPA and high E2 treated embryos for *SLC2A4* (*P* = 0.09) and *GPI* (*P* < 0.1).

### Experiment 2: Effects of oestrogen receptor (ER) inhibitor (fulvestrant) on embryo development

**Figure 5:**
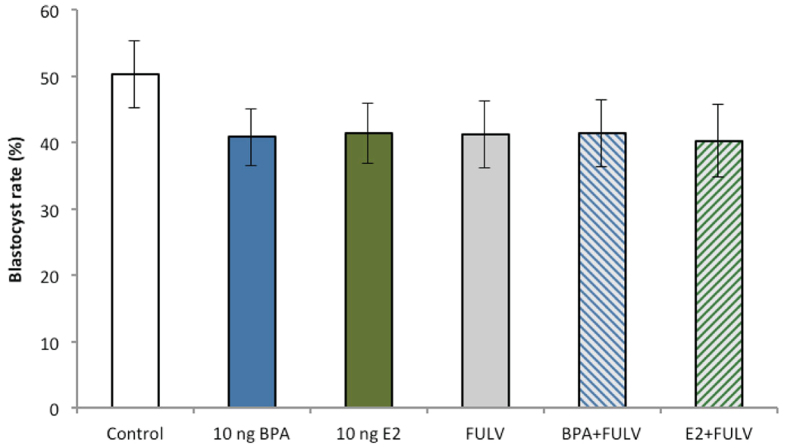
Blastocyst rates of control embryos or embryos treated with the ER inhibitor, fulvestrant (FULV, 10 ng/mL) in the presence or absence of BPA (10 ng/mL) or E2 (10 ng/mL) from day 3.5 to 7.5 of culture (≥180 8^+^ cell stage embryos per treatment across six biological replicates). Data are mean ± s.e.m.

**Figure 6:**
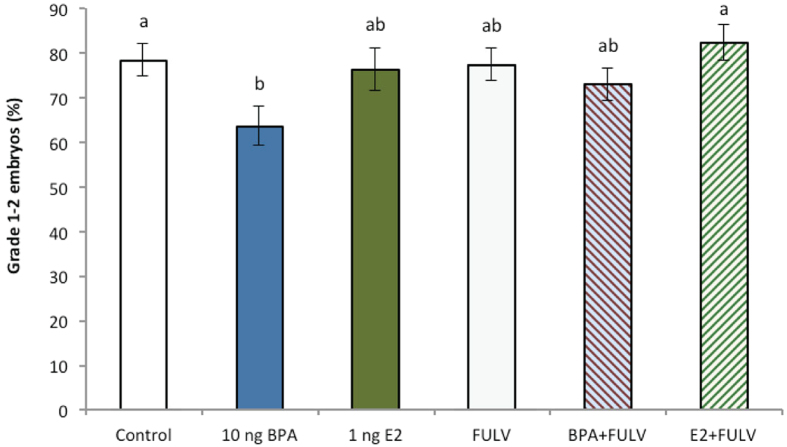
Effects of the ER inhibitor fulvestrant (FULV, 10 ng/mL) in the presence or absence of BPA (10 ng/mL) or E2 (10 ng/mL) from day 3.5 to 7.5 of culture on the percentage of grade 1 and 2 (transferrable quality) blastocysts (≥180 8^+^ cell stage embryos per treatment across six biological replicates). All data are mean ± s.e.m. Different superscript letters denote *P* < 0.05.

To determine whether the effects of BPA were mediated, at least in part, via oestrogen-regulated pathways, embryos were cultured in the high (10 ng/mL) BPA or E2 concentrations, based on the findings of *Experiment 1*, in the presence or absence of the ER inhibitor (fulvestrant, FULV). In total, ≥180 8^+^ cell stage embryos from six cultures were allocated into each group in *Experiment 2* (Control n = 195, BPA n = 225, E2 n = 225, FULV n = 195, BPA + FULV n = 240, E2 + FULV n = 180). No differences in blastocyst development were evident between any of the treatment groups (*P* > 0.1, Fig. 5), with development rates comparable to control and those in *Experiment 1*. Equally, no effects of any treatment were evident on the percentage of day 7.5 pre-expanded or post-expanded embryos compared with controls (data not shown, *P* > 0.1). Similar to *Experiment 1*, a decrease in the percentage of transferrable quality (grades 1–2) blastocysts was detected in embryos exposed to high BPA compared with control and E2 + FULV embryos (*P* < 0.05; Fig. 6). No differences were evident between other treatment groups (*P* > 0.1).

### Experiment 2: Effects of fulvestrant on blastocyst cell number and lineage allocation

Treatment of embryos in the presence of FULV, high BPA or high E2 with or without FULV did not result in any difference in blastocyst total cell number, cell lineage allocation (ICM or TE), their ratios or the percentage of ICM cells (Table 3, *P* > 0.1).

### Experiment 2: Effects of fulvestrant on embryo metabolism

**Figure 7:**
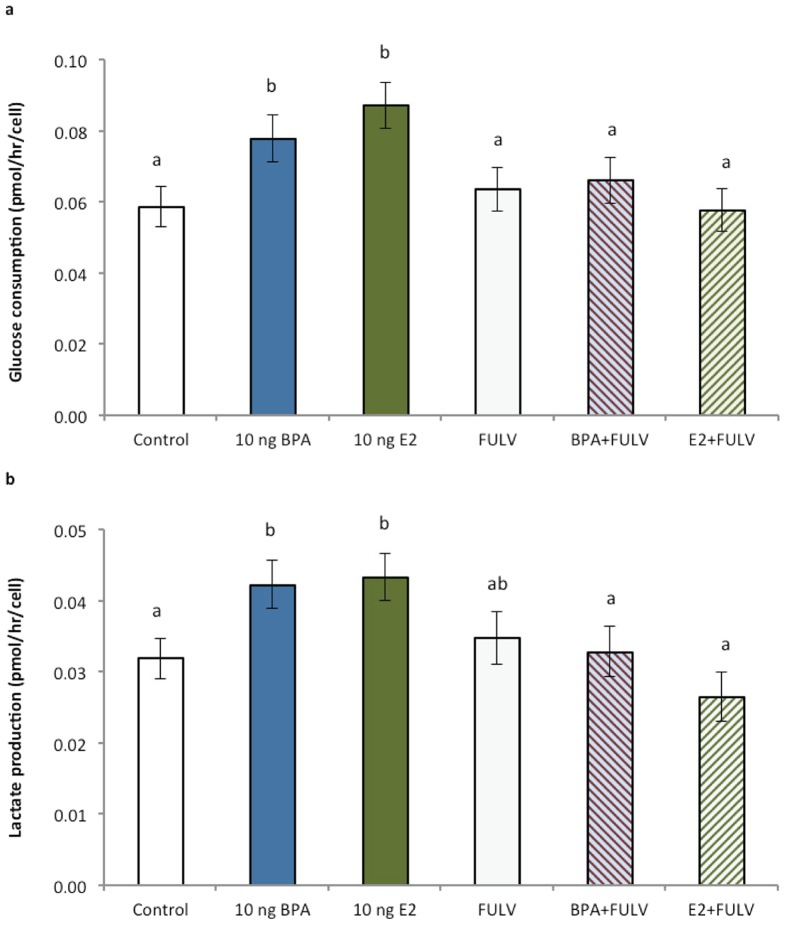
Effects of the ER inhibitor fulvestrant (FULV, 10 ng/mL) in the presence or absence of BPA (10 ng/mL) or E2 (10 ng/mL) from day 3.5 to 7.5 of culture on (**a**) glucose consumption (pmol/h/cell) and (**b**) lactate production of embryos (pmol/h/cell). n ≥ 25 embryos per group across six biological replicates. All data are mean ± s.e.m. Different superscript letters denote *P* < 0.05.

Similar to the results of *Experiment 1*, increased glucose consumption of individual blastocysts on a per cell basis was evident for embryos exposed to high BPA (*P* < 0.05) and high E2 (*P* < 0.01) compared to control (Fig. 7a,b). No change in glucose consumption or lactate production was observed between control embryos and any embryos cultured in the presence of the ER inhibitor (FULV; P > 0.1). Notably however, the increased glucose consumption and lactate production of embryos exposed to high BPA and high E2 was abrogated when cultured in the presence of FULV (BPA + FULV, *P* < 0.05; E2 + FULV, *P* < 0.01), to be no different to those of control embryos.

## Discussion

This study determined the effects of acute exposure, between days 3.5 to 7.5 post-fertilisation, to environmentally relevant BPA concentrations on bovine embryo development and metabolism. BPA at a high (10 ng/mL), yet still environmentally applicable concentration, reduced blastocyst development, and negatively impacted embryo quality, without a change in cell number or lineage allocation. Importantly, high BPA-exposed embryos also demonstrated an altered metabolic profile, with significantly increased glucose consumption, suggesting potential early perturbation of offspring metabolism. The negative development and metabolic effects determined in the high BPA-treated embryos were mirrored in embryos treated with the equivalent concentration of E2. Addition of the ER inhibitor fluvestrant however was able to negate all the developmental and metabolic effects induced by the environmentally relevant BPA concentration. This finding demonstrates that even acute BPA exposure early in embryo development can modulate developmental potential and metabolism. Moreover, this supports the hypothesis that BPA predominantly acts via oestrogen-mediated pathways.

The current finding of reduced blastocyst development following environmentally relevant BPA exposure (10 ng/mL) is consistent with other studies examining *in vitro* embryo development^33^,^34^,^35^, although previously 30 ng/mL BPA was used when the timing of exposure was limited to only oocyte maturation^33^,^34^. In the present study decreased development was evident with no differences in cell number or lineage allocation, with exposure to environmentally relevant, not pharmacological, BPA concentrations. Similar findings were also evident when bovine embryos were exposed to comparable concentrations (30 ng/mL)^33^,^34^, although this is higher than that considered as environmentally-relevant. In the present study, BPA supplementation resulted in a reduced number of bovine embryos reaching the blastocyst rate, and a lower percentage of transferrable quality embryos, that potentially suggests a negative impact on implantation success. Reduced implantation rates of embryos exposed to BPA via subcutaneous injection have been reported in mice^31^,^51^. These findings, together with perturbed embryonic glucose consumption, suggest BPA exposure may partially contribute to sub-fertility. Alternatively, in those embryos that do result in a live birth, these subtle changes may not manifest until offspring maturity, in the form of an increased pre-disposition to metabolic syndrome or obesity. Human epidemiological studies have identified an association with gestational BPA exposure and obesity^15^,^16^, as well as type II diabetes^17^, thus supporting this premise. Longitudinal studies following transfer of BPA exposed embryos will however be required to confirm this hypothesis.

Notably, the adverse effects of BPA treatment on bovine embryo development and quality were replicated in the presence of E2, suggesting that BPA may be predominantly acting via ER activation. Similar effects between BPA- and E2-treated embryos were evident in all parameters investigated including blastocyst rate, embryo quality and glucose consumption, which strongly supports the hypothesis that oestrogenic activities of BPA mediate effects in the bovine embryo. Furthermore, exposure of bovine embryos to the ER inhibitor, fulvestrant, negated the high BPA or high E2 effects on embryo development and increased glucose consumption. The current data thus provides evidence that BPA may elicit changes in development, quality and metabolism through oestrogen-regulated pathways, although alternative mechanisms cannot be excluded.

Associated with reduced development and quality, BPA exposure led to a significant increase in glucose consumption by bovine pre-implantation blastocysts. Glucose can be utilized by pathways other than glycolysis, such as the pentose-phosphate pathway (PPP), as reviewed in Wamelink^52^. The PPP plays an important role in normal bovine embryo function, as exposure of embryos to PPP inhibitor and/or glucose reduces blastocyst rate^43^,^44^. No changes were evident however in the expression of genes involved in PPP (*G6PD*, *PGLS*) or the other genes analysed in BPA-treated embryos. This finding is consistent with Ferris and colleagues^34^, who identified no expression change in ER-β, cell cycle or metabolic (including *SLC2A1* investigated here) genes of bovine blastocysts exposed to equivalent (15 mg/mL) BPA concentrations. Potentially this may be explained by the variability in embryo development stage and quality, as well as sex across treatments in the pooled samples. Despite this, expression appears to be increased, although not significantly, in the high BPA and high E2-treated embryos for *SLC2A4* (*GLUT4*), consistent with increased glucose transporter activity facilitating the increased glucose consumption identified with these treatments. For all the genes investigated it is anticipated a larger sample size or a longer duration of exposure may have resulted in significant differences being identified. Alternatively, BPA may regulate ERs at the protein or the post-translational level, rather than at the level of gene expression, supported by findings from our inhibitor studies. Moreover, epigenetic mechanisms, such as DNA methylation are also known to be modulated following BPA exposure^21^,^53^. Investigation of metabolic gene expression may also provide limited information or no differences, since these are independent to enzyme activity within metabolic pathways, such as the PPP. Hence further investigation of the molecular mechanisms involved is warranted. This is especially pertinent for understanding the potential for BPA and other endocrine disruptors in eliciting a trans-generational effect^1^,^4^.

One strength of the current study is that BPA exposure in culture media enables the direct effects of specific concentrations, and periods of exposure, on embryo characteristics to be investigated independent of indirect maternal environment effects. In addition, the current experimental design allowed the effects of BPA to be examined after embryonic genome activation, removing any inherent maternal carry-over effects. Use of the bovine model also removes the likelihood of prior exposure to BPA. In the current study, even though BPA exposure was acute, significant embryo development, quality and metabolic perturbations were evident. Perturbations in metabolism are consistently associated with alterations to embryo viability and long-term health, as reviewed by Leese^54^. Consequently, it is predicted *in vivo*, due to chronic exposure to BPA being present before and throughout gestation, even more pronounced effects may manifest in offspring and the mother; supported by studies in mice^19^,^21^,^55^. A further strength of this study is that the bovine embryo more closely resembles the human embryo compared with the mouse in many aspects, such as the timing of embryo genome activation^56^, microtubule pattern during fertilisation^57^, and the embryonic transcriptome^58^; advocating potentially improved translation of our findings to humans.

The current study has identified a detrimental effect of acute BPA exposure on the pre-implantation bovine embryo. Long-term studies with gestational exposure to higher BPA concentrations in mice and rats have determined increased body weight, serum insulin concentrations, as well as impaired glucose homeostasis in resultant adult offspring, demonstrating the ability of BPA to influence later life health^19^,^21^,^59^. BPA was also recently found to modify the milk lipid composition of rats, thus can also indirectly influence the growth of offspring^60^. Although the exact mechanism by which BPA affects an individual’s phenotype is still unclear, the current study provides evidence that BPA can induce altered metabolic profiles even at the early stages of development. Overall, the detrimental effects of BPA observed in this study as well others, in which BPA exposure extended through all stages of development, from gamete maturation through fetal development and into adulthood, emphasize the need for further research in this area and a review of BPA use in everyday products.

## Additional Information

**How to cite this article**: Choi, B.-I. *et al*. Bisphenol A affects early bovine embryo development and metabolism that is negated by an oestrogen receptor inhibitor. *Sci. Rep.* **6**, 29318; doi: 10.1038/srep29318 (2016).

## Figures and Tables

**Table 1:** Summary of qRT-PCR genes and primer details.

Gene	Primer sequence	Reference and/or Gene accession number	Product length (bp)
Oestrogen receptor α (*ERα*)			
* Forward*	5′-CACGATTGATAAAAACAGGAGGAA-3′	NM_001001443	108
* Reverse*	5′-CTCCCTCCTCTTCGGTCTTTC-3′		
Oestrogen receptor β (*ERβ*)			
* Forward*	5′-CATTGCCAGCCGTCAGTTCT-3′	NM_174051	120
* Reverse*	5′-GCTCCCACTAGCCTTCCTTTTC-3′		
Glucose transporter 1 (*SLC2A1*)			
* Forward*	5′-CTGATCCTGGGTCGCTTCAT-3′	NM_174602^61^	68
* Reverse*	5′-ACGTACATGGGCACAAAACCA-3′		
Glucose transporter 3 (*SLC2A3*)			
* Forward*	5′-GATGTCGCAGGAGAAGCAAGT-3′	NM_174603	98
* Reverse*	5′-GCTGGGAGAGCTGGAGCAT-3′		
Glucose transporter 4 (*SLC2A4*)			
* Forward*	5′-ATGGGTCCCTACGTCTTTCTTCT-3′	NM_174604	114
* Reverse*	5′-AACGGCTGAGATCTGGTCAAAC-3′		
Glucose-6-phosphate dehydrogenase (*G6PD*)			
* Forward*	5′-AGGCTGGAACCGCATCATC-3′	NM_001244135	103
* Reverse*	5′-GATCTGGTCCTCGTGGAACAG-3′		
Glucose-6-phosphate isomerase (*GPI*)			
* Forward*	5′-AAATCGCCCGACCAACTCT-3′	NM_001040471	100
* Reverse*	5′-GATGCCCTGAACGAAGATCTTG-3′		
Aconitase 2 (*ACO2*)			
* Forward*	5′-GGCAAGCCGCTGACATGTAT-3′	NM_173977	100
* Reverse*	5′-CGCGGAACCACTCGATCT-3′		
6-phosphogluconolactonase (*PGLS*)			
* Forward*	5′-ATTCTGGGTGTGGGTCCTGAT-3′	NM_001038580	109
* Reverse*	5′-GTGGTTTCGGAGAGTCACTGATG-3′		
Mitochondrial transcription factor A (*TFAM*)			
* Forward*	5′-CGACTGCGCTATCCCTTTAG-3′	NM_001034016	85
* Reverse*	5′-AAGTCATGGGCTTCTTTGGA-3′		
18S ribosomal RNA			
* Forward*	5′-AGAAACGGCTACCACATCCAA-3′	NR_036642^61^	91
* Reverse*	5′-CCTGTATTGTTATTTTTCGTCACTACCT-3′		

**Table 2:** Cell lineage allocation of blastocysts in control, BPA or E2 supplemented treatment groups (n = 98 embryos across five biological replicates).

Treatment	n	ICM	TE	TOTAL	ICM/TE	% ICM
Control	23	46.7 ± 3.4	102.4 ± 4.6	148.6 ± 7.1	0.46 ± 0.03	31.0 ± 1.2
1 ng/mL BPA	21	51.5 ± 4.9	105.4 ± 8.1	156.9 ± 12.4	0.49 ± 0.03	32.2 ± 1.3
10 ng/mL BPA	23	51.5 ± 3.2	100.5 ± 5.3	152.0 ± 7.8	0.52 ± 0.02	33.8 ± 1.1
1 ng/mL E2	14	51.2 ± 4.5	105.3 ± 4.8	156.5 ± 8.4	0.48 ± 0.03	32.1 ± 1.0
10 ng/mL E2	27	49.3 ± 2.7	101.9 ± 5.4	151.1 ± 7.5	0.50 ± 0.03	32.7 ± 1.1

ICM = inner cell mass and TE = trophectoderm cells. All data are means ± SEM.

**Table 3:** Cell lineage allocation of blastocysts in control, ER inhibitor fulvestant (FULV; 10 ng/mL) in the absence and presence of BPA (10 ng/mL) or E2 (10 ng/mL) (n = 59 embryos across three biological replicates).

Treatment	n	ICM	TE	TOTAL	ICM/TE	% ICM
Control	7	47.0 ± 4.8	92.0 ± 8.2	139.0 ± 12.1	0.51 ± 0.04	33.4 ± 2.6
BPA	10	51.6 ± 4.1	92.1 ± 6.9	143.7 ± 13.0	0.53 ± 0.03	32.3 ± 2.1
E2	9	51.3 ± 4.3	100.4 ± 7.3	151.8 ± 10.7	0.51 ± 0.03	33.7 ± 2.3
FULV	8	43.1 ± 4.5	93.3 ± 7.7	136.4 ± 13.1	0.48 ± 0.03	32.3 ± 2.4
BPA + FULV	9	43.0 ± 4.3	94.9 ± 7.3	137.9 ± 10.7	0.45 ± 0.03	30.9 ± 2.3
E2 + FULV	6	39.3 ± 5.2	95.3 ± 8.9	133.7 ± 13.1	0.43 ± 0.03	28.6 ± 2.8

ICM = inner cell mass and TE = trophectoderm cells. All data are means ± SEM.
